# Cyclosporin promotes neurorestoration and cell replacement therapy in pre-clinical models of Parkinson’s disease

**DOI:** 10.1186/s40478-015-0263-6

**Published:** 2015-12-14

**Authors:** Anna Tamburrino, Madeline J. Churchill, Oi W. Wan, Yolanda Colino-Sanguino, Rossana Ippolito, Sofie Bergstrand, Daniel A. Wolf, Niculin J. Herz, Michelle D. Sconce, Anders Björklund, Charles K. Meshul, Mickael Decressac

**Affiliations:** Telethon Institute of Genetics and Medicine (TIGEM), Pozzuoli, Italy; Veterans Hospital/Research Services/Portland and Department of Behavioral Neuroscience Oregon Health &, Science University, Portland, OR USA; Wallenberg Neuroscience Center, Department of Experimental Medical Sciences, Lund University, Lund, Sweden; Baylor College of Medicine, Jan and Dan Duncan Neurological Research Institute, Houston, USA

**Keywords:** Alpha-synuclein, Cyclosporin, Inflammation, Parkinson’s disease, Transplantation, Neurorestoration

## Abstract

**Background:**

The early clinical trials using fetal ventral mesencephalic (VM) allografts in Parkinson’s disease (PD) patients have shown efficacy (albeit not in all cases) and have paved the way for further development of cell replacement therapy strategies in PD. The preclinical work that led to these clinical trials used allografts of fetal VM tissue placed into 6-OHDA lesioned rats, while the patients received similar allografts under cover of immunosuppression in an α-synuclein disease state. Thus developing models that more faithfully replicate the clinical scenario would be a useful tool for the translation of such cell-based therapies to the clinic.

**Results:**

Here, we show that while providing functional recovery, transplantation of fetal dopamine neurons into the AAV-α-synuclein rat model of PD resulted in smaller-sized grafts as compared to similar grafts placed into the 6-OHDA-lesioned striatum. Additionally, we found that cyclosporin treatment was able to promote the survival of the transplanted cells in this allografted state and surprisingly also provided therapeutic benefit in sham-operated animals. We demonstrated that delayed cyclosporin treatment afforded neurorestoration in three complementary models of PD including the Thy1-α-synuclein transgenic mouse, a novel AAV-α-synuclein mouse model, and the MPTP mouse model. We then explored the mechanisms for this benefit of cyclosporin and found it was mediated by both cell-autonomous mechanisms and non-cell autonomous mechanisms.

**Conclusion:**

This study provides compelling evidence in favor for the use of immunosuppression in all grafted PD patients receiving cell replacement therapy, regardless of the immunological mismatch between donor and host cells, and also suggests that cyclosporine treatment itself may act as a disease-modifying therapy in all PD patients.

**Electronic supplementary material:**

The online version of this article (doi:10.1186/s40478-015-0263-6) contains supplementary material, which is available to authorized users.

## Introduction

The progressive degeneration of nigral dopamine neurons is a central element in the pathophysiology of Parkinson’s disease (PD). While no protective therapy has been identified, dopaminergic drugs offer a symptomatic relief to patients but over time these medications become less effective as the disease progresses. Over the last three decades, many studies have shown that replacement of lost dopamine neurons by transplantation of dopamine-producing cells into the diseased rodent brain is a promising avenue of research for PD [3, 4]. Among the various sources of cells tested to date, fetal dopaminergic progenitors derived from the developing ventral mesencephalon (VM) remain the best source of cells identified to date. These cells have entered clinical trials, and while the results are variable, it has been shown that in some patients there is substantial long-term benefit from such grafts [4, 19, 25, 32, 42, 58]. While the reported long-term clinical improvement seen in some patients clearly sustain the therapeutic potential of this approach, the mixed outcome of these trials has underscored the importance of patient selection, the need for a better standardization of the surgical procedure and optimization of the trial design [3].

These clinical studies have also underlined the limited predictability of the animal models of PD that were available at the time as well as the existence of additional factors which may have influenced the final clinical outcome [44]. All the pre-clinical studies performed prior to the clinical trials were conducted in toxin-based models of PD obtained by either injection of 6-hydroxydopamine (6-OHDA) in rats or 1-methyl-4-phenyl-1,2,3,6-tetrahydropyridine (MPTP) in non-human primates [4–6, 8, 51]. Although these models are useful for looking at the restoration of the dopaminergic striatal innervation, they fail to replicate important pathological features including the development of the alpha-synuclein (α-synuclein) aggregation seen in the PD brain, which may influence the integration and long-term survival of the transplanted cells. In addition, neurotoxic lesions are acute ways of damaging the dopaminergic system whilst the PD pathology is progressive. Finally, most rodent studies utilize isogenic grafts (i.e. tissue from inbred strains) while patients have received allografted tissue, thereby making the immune response an important modifier of the clinical outcome.

In order to overcome some of the limitations of the neurotoxin models of PD, attempts have been made to create new models which replicate the progressive nature of the pathology and its α-synuclein basis [55], but this has not really been done to any great extent to look at the survival, integration and functional impact of fetal VM dopaminergic grafts. The relevance of further research in this area is supported by the recent observations of Lewy body-like pathology in donor-derived dopamine neurons grafted into subjects with PD, suggesting a mechanism of host-to-graft disease propagation [28, 31] and thereby demonstrating that the α-synuclein pathology of the host brain can affect the grafted cells themselves. In vitro studies provided the first evidence for the existence of a “prion-like” propagation of aggregated pathogenic α-synuclein, which was later observed in rodents and non-human primates [18, 33–35, 45, 57]. The recent development of a rodent model of PD based on targeted overexpression of human α-synuclein using intra-nigral injection of an adeno-associated viral vector (AAV) has opened the possibility to investigate in more detail the graft effects in the host (as opposed to the host on the graft) and have done so by conducting side-by-side comparative studies with the standard 6-OHDA-induced rat model of PD.

Here we demonstrate that such grafts induce a sustained low-grade level of inflammation and that this immune response negatively impacts on the survival of the grafted neurons. Furthermore we provide evidence that immunosuppression using cyclosporin A (CsA) not only promoted the long-term survival of the transplanted cells but also afforded a restorative effect in several different models of PD based on α-synuclein or MPTP toxicity. This therapeutic effect resulted from a combination of non-cell autonomous and cell autonomous mechanisms, and not just through its immunosuppressive effects.

## Material and methods

### Primary cultures of cortical neurons

Primary neuronal cultures were prepared from the E16 C57BL/6 mouse brain. Dissociated cortical neurons were plated onto 6-well plates at a density of 50,000 cells/cm^2^. Treatment with CsA (10 μM) was performed at 7–9 days in vitro and analyses were carried out 24 h later.

### Viral vectors

Production of the AAV2/6 vector expressing the human wild-type α-synuclein under the control of the human synapsin-1 promoter and enhanced using a woodchuck hepatitis virus post-transcriptional regulatory element was performed as previously described (Decressac et al. 2012). Genome copy (gc) titer was determined by real-time quantitative PCR and the following vector concentration was used: 1.6 × 10^12^ gc/ml.

The Cre-regulated AAV-α-synuclein vector (thereafter referred as AAV-FlexOFF-α-synuclein) was generated by inserting the cDNA of wild-type human α-synuclein between two pairs of heterotypic, antiparallel loxP sequences [2]. This vector design results in flipping and inactivation of the transgene specifically in Cre-expressing cells. Validation of the inactivation of the transgene in dopamine neurons was confirmed by injection of the AAV vector (6.8 × 10^9^ gc/ml) in the ventral midbrain of Dat-Cre mice (Additional file 1: Supplementary Figure 1) and a similar dose was used for experiments in Dat-Cre^ERT2^ mice.

### Animals

Three-month-old, adult female Sprague Dawley rats (Charles River), 225 − 250 g at the time of surgery, were housed two to three per cage. Adult C57Bl/6, DAT-IRES-Cre (stock number 006660, Jackson Laboratories) and DAT-Cre^ERT2^ mice (stock number 016583, Jackson Laboratories), aged 12 weeks old at the time of AAV virus injection, were housed two to six per cage. In both Cre transgenic mice, expression of the Cre recombinase is under control of the promoter of the dopamine transporter (DAT) and hence is detected in dopaminergic neurons since embryonic stage. While the enzyme can freely translocate to the nucleus to exert its activity in DAT-IRES-Cre mice, its function is conditioned by exposure to synthetic ligands such as tamoxifen in the DAT-Cre^ERT2^ mice since the enzyme is fused to a mutant variant of the human estrogen receptor.

Ten-month-old transgenic mice overexpressing wild-type human α-synuclein under the neuron-specific Thy1 promoter (“Line 61”; provided by Pr. E. Masliah, University of California) were housed under similar conditions [48]. All animals had *ad libitum* access to food and water during a 12 h light/dark cycle.

All procedures were conducted in accordance with guidelines set by the local Ethical Committees for the use of laboratory animals (Lund-Malmo region and Naples), the European Directives (2010/63/EU), and the federal guidelines of the Public Health Service Policy on the Humane Care and Use of Laboratory Animals.

### Lesion procedures

In rats, all surgical procedures were performed under general anesthesia using a 20:1 mixture of fentanylcitrate (Fentanyl) and medetomidin hypochloride (Dormitor) (Apoteksbolaget, Sweden), injected intraperitoneally (i.p). Solutions containing the AAV-α-synuclein vector or 6-OHDA were injected using a 10 μl Hamilton Neuros syringe. Rats received either 3 μl of AAV-α-synuclein into the substantia nigra (SN) or 3 μl of 6-OHDA (3.5 μg/μl free base dissolved in a solution of 0.2 mg/ml L-ascorbic acid in 0.9 % w/v NaCl) into the medial forebrain bundle (MFB). Infusions were performed at a rate of 0.2 μl/min and the needle was left in place for an additional 3 min period before being slowly retracted. Injections were carried out unilaterally on the right side, at the following coordinates (flat skull position) for the SN: antero-posterior: −5.3 mm, medio-lateral: −1.7 mm, dorso-ventral: −7.2 mm below dural surface; and for the MFB: antero-posterior: −4.4 mm, medio-lateral: −1.1 mm, dorso-ventral: −7.8 mm below dural surface, calculated relative to bregma according to the stereotaxic atlas of Paxinos and Watson [43].

In mice, surgical procedures were performed under general anesthesia using isoflurane. A 5 μl Hamilton syringe was used to inject 1 μl of AAV vector in the mouse SN at the following coordinates: antero-posterior: −2.7 mm, medio-lateral: −1.2 mm, dorso-ventral: −4.2 mm below dural surface.

### Tamoxifen administration

Tamoxifen (Sigma, T-5648) was dissolved in corn oil (Sigma, C-8267) and ethanol in a 9:1 mixture at a final concentration of 10 mg/ml. New mixture was prepared every other day.

Eight weeks after AAV vector injection, Dat-Cre^ERT2^ mice were tested for motor performance and manifest animals received i.p injections of 2 mg of tamoxifen per day for 5 consecutive days to inactivate the transgene in dopamine neurons.

### Transplantation procedure

VM from E14 rat embryos were dissected as previously described [10], incubated in Dulbecco’s modified eagle medium (DMEM, Invitrogen) containing 0.1 % trypsin (Sigma-Aldrich) and 0.05 % DNase (Sigma-Aldrich) for 20 min at 37 °C and mechanically dissociated into a single cell suspension. After centrifugation (500 *g*, 5 min, 4 °C), the number of viable cells was estimated by trypan blue staining (Sigma-Aldrich). Cells were then re-suspended in DMEM/DNase at a concentration of 150,000 cells/μl and kept at room temperature until grafted.

Transplantation was performed under general anesthesia as described above. About 150,000 cells were injected as two 0.75 μl deposits in the lesioned striatum at the following coordinates: antero-posterior: +0.6 mm, medio-lateral: −3.1 mm, dorso-ventral: −4.5/-3.5 mm below the dural surface. Rats assigned to the sham control group received a similar injection of the vehicle solution only. Leftover cells were subject to an estimation of cell viability using trypan blue exclusion and survival was >95 %.

### Cyclosporin a treatment

For the transplantation experiments, rats received daily i.p injections of cyclosporin A (CsA) (10 mg/kg) or vehicle solution starting 2 days before transplantation and until the endpoint of the experiment [8, 21].

For the experiments in the AAV-FlexOFF-α-synuclein mouse model, animals received i.p injections of CsA (20 mg/kg, Sigma-Aldrich) every other day from the day following the last injection of tamoxifen and until the time of sacrifice.

For the experiments in the Thy1-α-synuclein mice, 10-month-old transgenic mice and wild-type littermates received daily i.p injections of CsA (20 mg/kg) or vehicle solution for 6 weeks.

The dose of CsA used in mice was calculated as follow, based on the standard dose used for the rats [30] and Body Surface Area (BSA) conversion (values from FDA Draft guidelines): dose in mice (mg/kg) = dose in rats (mg/kg) × (mouse Km/rat Km), where K_m_ factor = Weight (kg)/Body Surface area (m^2^); for mice: weight = 0.02 kg, BSA = 0.007 and K_m_ = 3; for rats: weight = 0.15 kg, BSA = 0.025 and K_m_ = 6. All behavioural tests were performed at least 2 h after CsA injection.

### Protocols for MPTP and CsA injections

C57BL/6 J mice (Jackson Laboratories, Bar Harbor, ME, USA) (8 weeks old) were injected with increasing doses of MPTP (8 mg/kg, 10 mg/kg, 24 mg/kg and 32 mg/kg, 5 days/week, i.p.) for a total of 4 weeks (*30*). Three days following MPTP, mice were administered either vehicle or CsA at a concentration of 20 mg/kg in normal saline for 4 weeks (5 days /week, i.p.).

### Estimation of transplants volume

The volume of the graft was calculated from TH-stained striatal sections using the Cavalieri formula taking into account the sum of all the areas and correcting for section thickness and sample frequency [Volume (mm^3^) = Sum of areas (mm^2^) × 35 μm × 6 series] [30].

### ELISA analysis

Striata were dissected and homogenized in TPER lysis buffer containing phosphatase cocktail I and II (1:100; Sigma-Aldrich). Samples were analyzed for the level of cytokines (Pierce, ThermoFischer Scientific).

### Statistical analysis

Statistical analyses were conducted using the GraphPad Prism software (version 6.0f) or the Jmp 11 software (SAS). Unpaired two-tailed Student's *t* tests or one-way ANOVA tests with Dunnett's multiple comparison test were performed to analyze the difference between experimental and control groups. Two-way repeated measures ANOVA tests were used to detect interactions between time and treatment. The data were collected and processed in a randomized and blinded manner. No statistical methods were used to predetermine sample size, but our sample sizes are similar to those generally employed in the field. All values are presented as mean ± standard error of the mean. Statistical significance was set at *P* < 0.05.

Behavioural tests, histological procedures, cell counting, optical densitometry analysis and western blot protocols are described in details in Additional file 2: Supplementary Information.

## Results

### Alpha-synuclein in the host striatum affects the long-term survival of grafted VM transplants

We first explored the survival of fetal dopamine neurons transplanted into the toxin-induced versus the α-synuclein based rat model of PD and their impact on motor function. Animals were lesioned by receiving either a unilateral injection of 6-OHDA into the MFB or an intra-nigral injection of an AAV-α-synuclein vector. Eight weeks later, when marked behavioral impairments were observed in both models using the cylinder and amphetamine-induced rotation tests, rats were assigned to receive either an intra-striatal graft of mesencephalic progenitors or sham surgery and their motor performance was monitored up to 12 weeks post-transplantation.

**Fig. 1:**
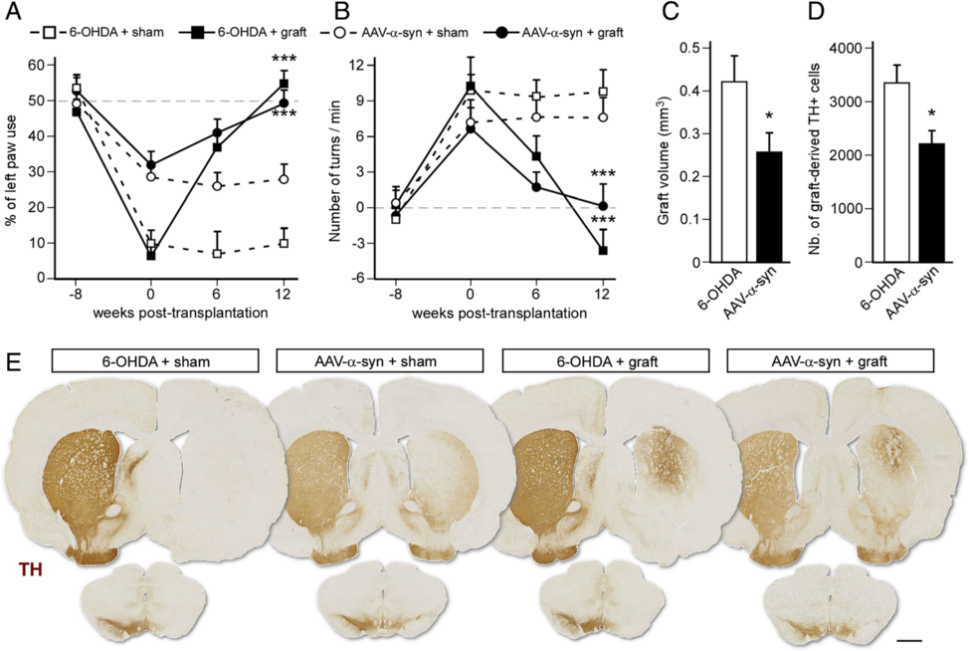
Survival and functional impact of mesencephalic grafts in the 6-OHDA and AAV-α-synuclein rat models. **a**-**b** Motor function was assessed before injection of an AAV-α-synuclein vector or 6-OHDA (−8 weeks time-point), before transplantation (0 week time-point), and 6 and 12 weeks post-transplantation using the cylinder test **a** and the amphetamine-induced rotation test (**b**). Data are expressed as mean ± SEM (*n* = 8-10 per group). ***P < 0.001 compared to the respective sham control group (*t*-test). **c**-**d** Assessment of graft volume (**c**) and quantification of the number of graft-derived TH-positive cells (**d**) showed a reduced survival of the transplant in the AAV-α-synuclein rat model. Data are expressed as mean ± SEM (*n* = 10-12 per group). *P < 0.05 (*t*-test). **e**: Forebrain (upper) and midbrain (lower) sections from all experimental groups were stained for TH to analyze the extent of the lesion and the survival of grafted dopamine neurons. Scale bar: 1.5 mm

We found that neural grafting provided an efficient and full recovery of motor function in both models and in both tests. As previously described, 6-OHDA lesioned rats showed no sign of a motor deficit on the cylinder test 12 weeks after transplantation (*P* < 0.001 compared to 6-OHDA + sham group) [F_3,42_ = 28.33; 2-way ANOVA treatment × time interaction, *P* < 0.001] and elicited an overcompensation in the rotation test (−3.6 ± 1.3 turns/min; *P* < 0.001 compared to 6-OHDA + sham group) [F_3,42_ = 26.93; 2-way ANOVA treatment x time interaction, *P* < 0.001] (Fig. 1a-b). Likewise, in the AAV-α-synuclein model, transplantation of fetal mesencephalic cells fully restored the behavioral deficit in the cylinder test (*P* < 0.001 compared to AAV-α-synuclein + sham group) (F_3,54_ = 9.83; 2-way ANOVA treatment × time interaction, *P* < 0.001) and in the rotation test [F_3,54_ = 8.03; 2-way ANOVA treatment × time interaction, *P* < 0.001], albeit with no overcompensation in this case (0.1 ± 0.15 turns/min; *P* < 0.001 compared to AAV-α-synuclein + sham group) (Fig. 1a–b).

Post-mortem analysis, however, revealed that the survival of the grafts was reduced in the AAV-α-synuclein model as compared to the 6-OHDA group. Estimation of the volume showed that transplants were significantly smaller in the AAV model (0.264 ± 0.038 mm^3^) as compared to the toxin model (0.425 ± 0.052 mm^3^; *P* < 0.05) (Fig. 1c, e). In parallel, stereological quantification revealed that grafts in the AAV-α-synuclein group contained fewer TH+ neurons (2139 ± 241 cells) compared to the 6-OHDA group (3270 ± 344 cells, *P* < 0.05) (Fig. 1d–e). Since striatal denervation in this model is not complete, it was not possible to distinguish between host-derived and graft-derived dopaminergic fibers and therefore to measure the degree of innervation originating from the transplanted cells.

### Alpha-synuclein overexpression in the striatum induces inflammation in vivo

Although functional recovery was observed in both rat models, we wanted to better understand the reasons for the reduced survival of grafted neurons in the AAV-α-synuclein model. Previous results obtained in the 6-OHDA rat model showed that the density of remaining striatal dopaminergic innervation can impact on the survival of the dopamine cells in the transplant [27]. As we previously described, 6-OHDA injection into the MFB results in a more complete loss of dopamine in the striatum compared to nigral injection of AAV-α-synuclein as seen by the greater loss of nigral dopamine neurons (3.1 ± 1.1 % cells for the 6-OHDA group vs. 32.7 ± 4.9 % for the AAV-α-synuclein group, *P* < 0.001) and striatal terminals (2.2 ± 0.6 % for the 6-OHDA group vs. 36.8 ± 3.4 % for the AAV-α-synuclein group, *P* < 0.001) (Fig. 1e). Therefore, the incomplete striatal denervation obtained in the AAV-α-synuclein model may account for the difference in survival of the graft-derived TH-positive neurons.

Other important phenomena may also have contributed to the reduced graft survival. Cell-to-cell transfer of α-synuclein has been well documented in in vitro and in vivo models [1, 18, 23, 29, 33–35, 57] as well as in the brains of grafted PD patients [28, 31]. However, this process is slow, affects only around 5 % of the transplanted cells and the impact on the survival of grafted neurons remains uncertain. Thus, we reasoned that, the host-to-graft transmission of α-synuclein is unlikely to explain the difference in the survival of graft-derived TH+ neurons and thus cannot be a major culprit.

**Fig. 2:**
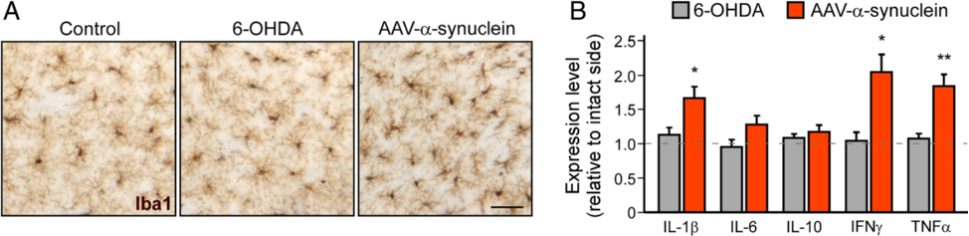
Alpha-synuclein overexpression induces long-term inflammation in the striatum. **a** Immunohistochemical staining of striatal sections against Iba1 8 weeks after 6-OHDA injection or α-synuclein overexpression. Scale bar: 40 μm. **b** Striatal levels of IL1b, IL6, IL10, TNFα and IFNγ were measured by ELISA 8 weeks after 6-OHDA MFB lesion or intra-nigral injection of an AAV-α-synuclein vector. Cytokine levels are expressed relative to the control side. Data are expressed as mean ± SEM (*n* = 4 per group). *P < 0.05 and **P < 0.01 compared to 6-OHDA group (*t*-test)

We also hypothesized that α-synuclein may affect neuronal survival in a non-cell autonomous manner by triggering inflammation. It is known that inflammation is a characteristic feature of the PD brain and of lesion models of PD, and previous studies in rodents have shown that a sustained, low-grade immune response impairs long-term survival and function of transplanted dopamine neurons [24, 50]. Staining of Iba-1+ microglia demonstrated sustained inflammation in the striatum of rats injected with the AAV-α-synuclein vector, while rats lesioned with 6-OHDA did not show such a response 8 weeks post-lesion (Fig. 2a). This observation was confirmed by measuring the levels of cytokines in the striatum. We detected that the pro-inflammatory factors IL-1β (*P* < 0.05), IFNγ (*P* < 0.05) and TNFα (*P* < 0.01) were significantly elevated in the striatum of rats over-expressing human α-synuclein (Fig. 2b). Inflammation in the AAV-based model was not compared to an AAV-GFP control group since the goal of the study was to study this process in degenerative conditions. In addition, previous work have demonstrated that the inflammatory response in this model is driven by α-synuclein toxicity and not by the viral vector [13].

### Cyclosporin treatment promotes graft survival and recovery in both grafted animals and in non-grafted animals lesioned with AAV-α-synuclein

While immunosuppression is an important requirement to prevent rejection and promote optimal graft survival in the context of peripheral allogeneic transplantation, it has not been systematically applied in all clinical trials assessing cell therapy in PD. Indeed there has been a debate as to whether this may have been one of the critical factors contributing to the mixed outcomes in the published clinical trials using fetal VM transplantation to treat PD [3]. In addition, although the etiology of PD remains largely unknown, a large body of evidence has led to the “multiple-hit” hypothesis where inflammation contributes to the progression of the disease [53].

To address the importance of inflammation and immunosuppression for cell replacement therapy in the AAV-based model, we performed a second set of experiments. In these experiments, rats received either an immunosuppressive regimen (daily i.p. injection of CsA, 10 mg/kg) or vehicle treatment 8 weeks after intra-nigral injection of AAV-α-synuclein and 3 days before receiving either a neural graft or sham surgery. Animals were tested for motor behavior 6 and 12 weeks post-transplantation while being maintained under immunosuppression or vehicle treatment.

**Fig. 3:**
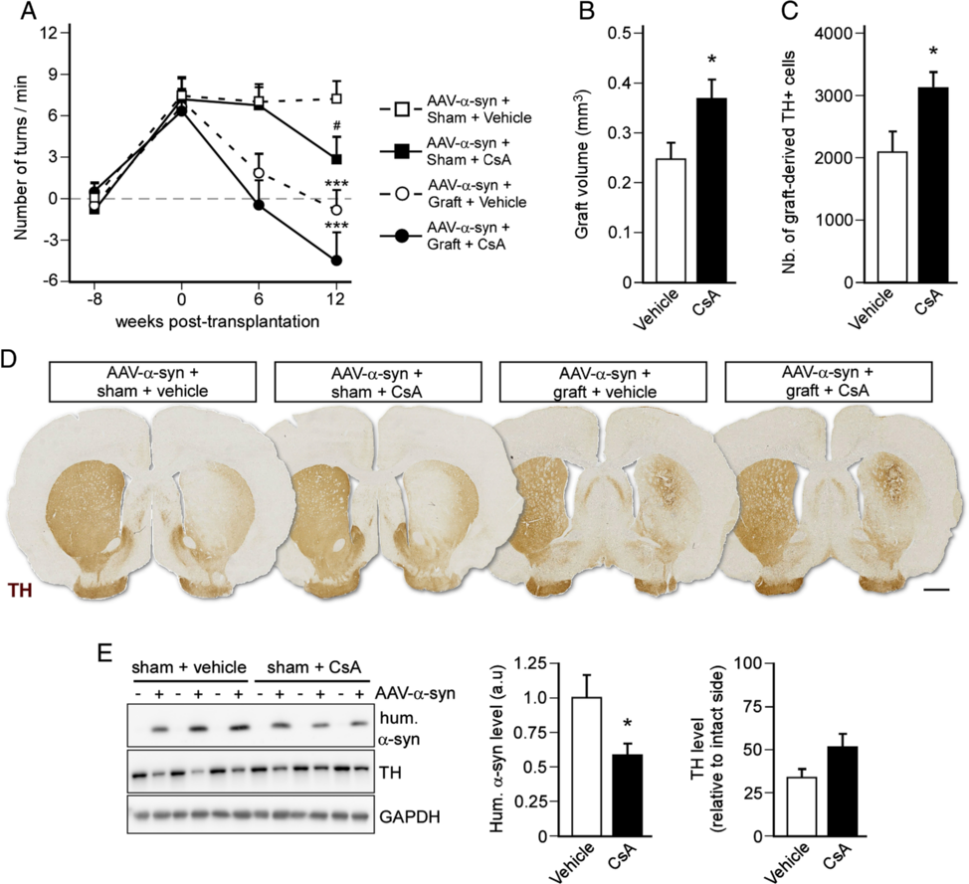
Cyclosporin treatment improves graft survival and reduces pathology in ungrafted AAV-α-synuclein lesioned rats. **a** Motor function was assessed before injection of an AAV-α-synuclein (−8 weeks time-point), before transplantation (0 week time-point), and 6 and 12 weeks post-transplantation using the amphetamine-induced rotation test. Data are expressed as mean ± SEM (*n* = 8-10 per group). ^#^ *P* < 0.05 compared to the respective vehicle treated group, ***P < 0.001 compared to the respective sham control group (*t*-test). **b**-**c** Assessment of graft volume (**b**) and quantification of the number of graft-derived TH-positive cells (**c**) showed increased survival of the transplant in the CsA treated group. Data are expressed as mean ± SEM (*n* = 8-10 per group). *P < 0.05 (*t*-test). **d** Striatal sections from all experimental groups were stained for TH to analyze the extent of the lesion and the survival of grafted dopamine neurons. Scale bar: 1.5 mm. **e** Western blot analysis of the striatal levels of human α-synuclein and TH in AAV-α-synuclein + sham rats treated with CsA (i.p., 10 mg/kg, daily) or vehicle. Data are expressed as mean ± SEM (*n* = 6 per group). *P < 0.05 (*t*-test)

Our results showed that immunosuppression had no effect on the speed of functional recovery [F_2,32_ = 1.10; 2-way ANOVA treatment x time interaction, *P* = 0.34] and did not statistically affect the magnitude of behavioral restoration (*P* = 0.08 compared to vehicle-treated animal at 12 weeks) (Fig. 3a), although we now saw the overcompensation on the rotational test (−4.3 ± 1.6 contralateral turns/min), similar to that we had observed in the 6-OHDA model (Fig. 1b). Consistent with these observations, morphological analysis showed that CsA treatment resulted in larger-sized grafts (0.369 ± 0.036 mm^3^ in the CsA-treated group; 0.246 ± 0.039 mm^3^ in the vehicle-treated group; *P* < 0.05) (Fig. 3b) with an increased number of graft-derived dopamine neurons (3116 ± 274 TH+ cells in the CsA-treated group; 2092 ± 314 TH+ cells in the vehicle treated group; *P* < 0.05) (Fig. 3c).

In addition, CsA treatment also induced a partial alleviation of the motor deficit in sham-operated animals (3.3 ± 1.2 turns/min) as compared to vehicle-treated animals (7.2 ± 1.0 turns/min, *P* < 0.05) (Fig. 3a). This observation prompted us to further explore substrates correlated with this improvement and western blot analysis revealed that the striatal level of human α-synuclein was reduced by CsA treatment (*P* < 0.05) (Fig. 3e) suggesting that this drug exert other actions in the brain besides immunosuppression. Although not being statistically significant, the level of TH was higher in immunosuppressed animals (*P* = 0.11), which may have explained the partial functional improvement we observed (Fig. 3e).

### Cyclosporin rescues motor and cognitive deficits in a range of α-synuclein models of Parkinson’s disease

The results found in the rats overexpressing α-synuclein treated with CsA prompted us to investigate the disease-modifying effect of this FDA-approved drug in complementary models of PD. The relevance of the current animal models obtained by α-synuclein overexpression is often criticized on the basis of the sustained supra-physiological expression of the transgene at levels that are higher than that seen even in genetic PD patients with triplication of the *SNCA* gene. To overcome this limitation, we developed a novel mouse model of PD which was engineered to allow for the spatial and temporal control of α-synuclein overexpression. We generated a genetic construct where the wild-type human α-synuclein cDNA is inserted between two pairs of heterotypic, antiparallel loxP sequences. This vector design results in flipping and inactivation of the transgene specifically in Cre-expressing cells (thereafter named FlexOFF-α-synuclein) (Additional file 1: Supplementary Figure 1A). To test the specificity of this construct, DAT-IRES-Cre mice received an intra-nigral injection of the AAV-FlexOFF-α-synuclein vector or a standard AAV-α-synuclein vector where transgene expression was driven by the synapsin1 promoter in both vectors [16]. Four weeks after AAV-FlexOFF-α-synuclein vector delivery, histological analysis revealed that essentially no dopaminergic neurons expressed the transgene while surrounding TH-negative neurons showed high levels of transgenic α-synuclein expression (Additional file 1: Supplementary Figure 1B). In contrast, human α-synuclein expression was observed in both dopaminergic and non-dopaminergic neurons after delivery of the AAV-α-synuclein vector (Additional file 1: Supplementary Figure 1B). Stereological analysis showed that inactivation of the transgene was associated with the absence of toxicity to the nigral dopamine neurons (Additional file 1: Supplentary Figure 1C) (*P* < 0.01).

**Fig. 4:**
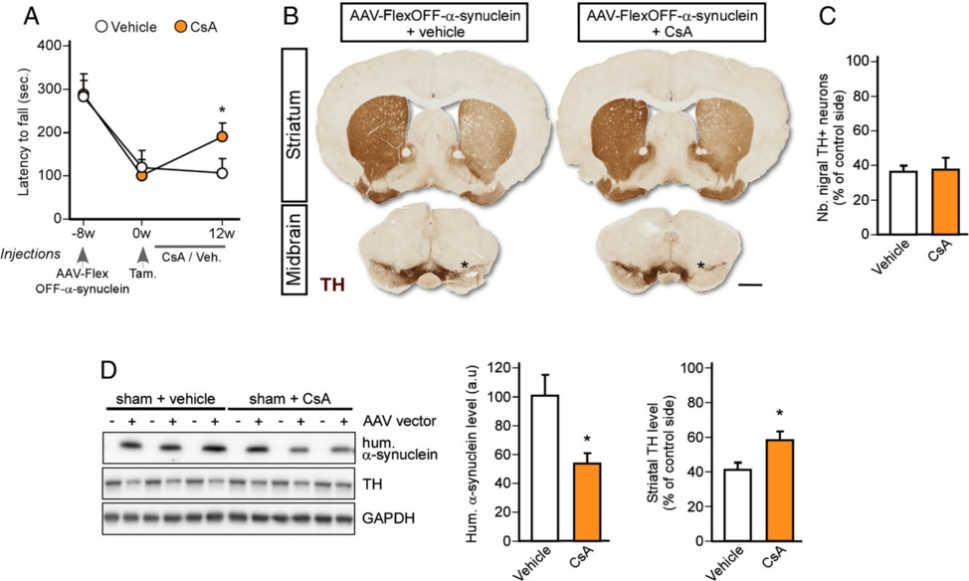
Cyclosporin treatment alleviates motor features in a novel α-synuclein-based model of PD. **a** Motor function was examined using the hanging wire test in AAV-FlexOFF-α-synuclein vector injected mice treated with vehicle solution or CsA (i.p., 20 mg/kg, 5 days/week). Data are expressed as mean ± SEM (*n* = 15 per group). *P < 0.05 (*t*-test). Tam: tamoxifen. **b** Striatal and midbrain sections stained for TH. Scale bar: 1 mm. Asterisk on midbrain sections indicates the site of injections of the AAV vector. **c** Stereological estimation of the number of TH-positive nigral neurons. Data are expressed as mean ± SEM (*n* = 10 per group). **d** Western blot analysis of the striatal levels of human α-synuclein and TH levels in both experimental groups. Data are expressed as mean ± SEM (*n* = 5 per group). *P < 0.05, compared respective vehicle group (*t*-test)

To test the restorative effect of CsA in this new model, DAT-Cre^ERT2^ mice received an intra-nigral injection of AAV-FlexOFF-α-synuclein vector. Eight weeks later, manifest mice were treated with tamoxifen so as to “switch off” the expression of human α-synuclein in dopamine neurons, and were then randomly assigned to receive either daily injections of CsA or vehicle solution. Our results indicate that, whilst vehicle-treated mice retained behavioural deficit 3 months after transgene inactivation, mice treated with CsA improved their motor performance (*P* < 0.05) (Fig. 4a). Histological analysis showed that CsA treatment did not affect the survival of nigral dopamine neurons (*P* = 0.82) (Fig. 4b-c), but as we found in the AAV rat model of PD (Fig. 3e), analysis of striatal tissue showed a reduction in human α-synuclein levels (*P* < 0.01) and an increased expression of TH (*P* < 0.05) (Fig. 4e).

**Fig. 5:**
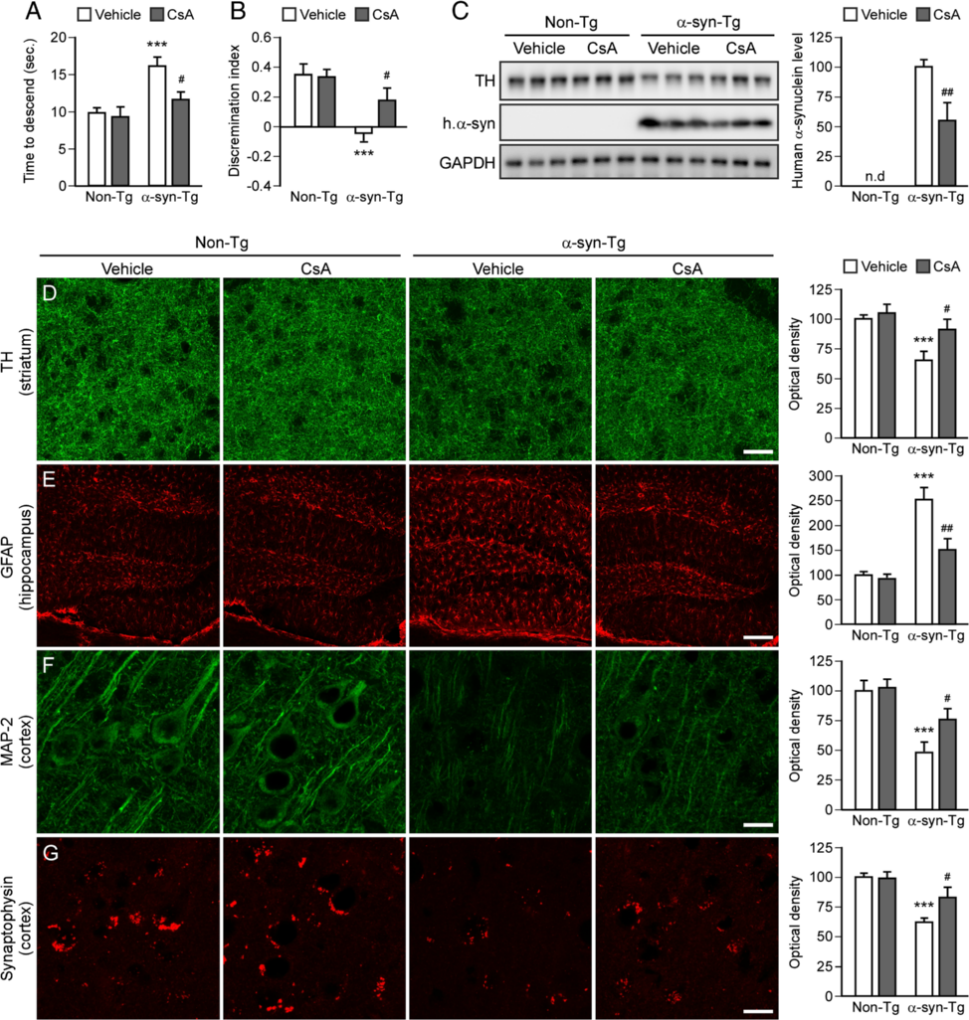
CsA treatment reverses pathology in the Thy-1-α-synuclein transgenic mouse. **a** Motor function was assessed using the pole test in 12 month old non-transgenic (non-Tg) and α-synuclein transgenic mice (α-syn-Tg) treated for 8 weeks with vehicle solution or CsA (i.p., 20 mg/kg, 5 days/week). Results are presented as the total time (time to turn + time to descend the pole). Data are expressed as mean ± SEM (*n* = 8-10 per group). ***P < 0.001 compared to respective non-Tg group, ^#^ *P* < 0.05 compared to respective vehicle treated group (one-way ANOVA). **b**: Cognitive function was examined using the novel object recognition test. The discrimination index was calculated based on 2 sessions performed 1 h apart. Data are expressed as mean ± SEM (*n* = 8-10 per group). ***P < 0.001, (one-way ANOVA). **c**: Western blot analysis of striatal human α-synuclein and TH expression in non-transgenic and transgenic mice treated with vehicle solution or CsA (i.p., 20 mg/kg, 5 days/week for 8 weeks). Data are expressed as mean ± SEM (*n* = 6 per group). ^##^ *P* < 0.01 compared to respective vehicle treated group (*t*-test). **d**-**g**: Immunofluorescence staining of TH in the striatum (**d**), GFAP in the hippocampus (**e**), and MAP-2 (**f**) and synaptophysin (**g**) in the cortex of all experimental groups. Scale bar: 150 μm (**d**-**e**), 10 μm (**f**-**g**). Quantification of immunofluorescence intensity in the neuropil. Data are expressed as mean ± SEM (*n* = 6-8 per group). ***P < 0.001 compared to respective non-Tg group, ^#^ *P* < 0.05 and ^##^ *P* < 0.01 compared to respective vehicle treated group (one-way ANOVA)

We then investigated whether CsA treatment could exert a restorative action in a transgenic mouse model of Parkinson’s disease whereby the expression of wild-type human α-synuclein is driven by the neuronal promoter Thy-1 [48]. Despite failing to elicit overt neurodegeneration, this mouse model develops intra-neuronal α-synuclein aggregates distributed throughout the brain with neuroinflammation and age-dependent motor and cognitive deficits [12]. These pathological features make this mouse an attractive model to study the effect of pharmacological interventions. Ten-month-old mice received daily i.p injections of CsA (20 mg/kg) for 6 weeks and were examined for behavioral deficits. Transgenic mice treated with CsA displayed enhanced motor and cognitive function (Fig. 5a–b) (Ps < 0.05). Post-mortem analysis again revealed that CsA treatment lowered the striatal level of human α-synuclein (*P* < 0.01) (Fig. 5c) and partially restored the level of TH (*P* < 0.05) (Fig. 5d). Densitometry measurements indicated that glial response was diminished in the hippocampus and that the cortical levels of synaptic markers MAP-2 and synaptophysin were partially restored in transgenic mice upon CsA treatment (Fig. 5e–g) (Ps < 0.05).

### Cyclosporin alleviates pathology in the MPTP mouse model of PD

**Fig. 6:**
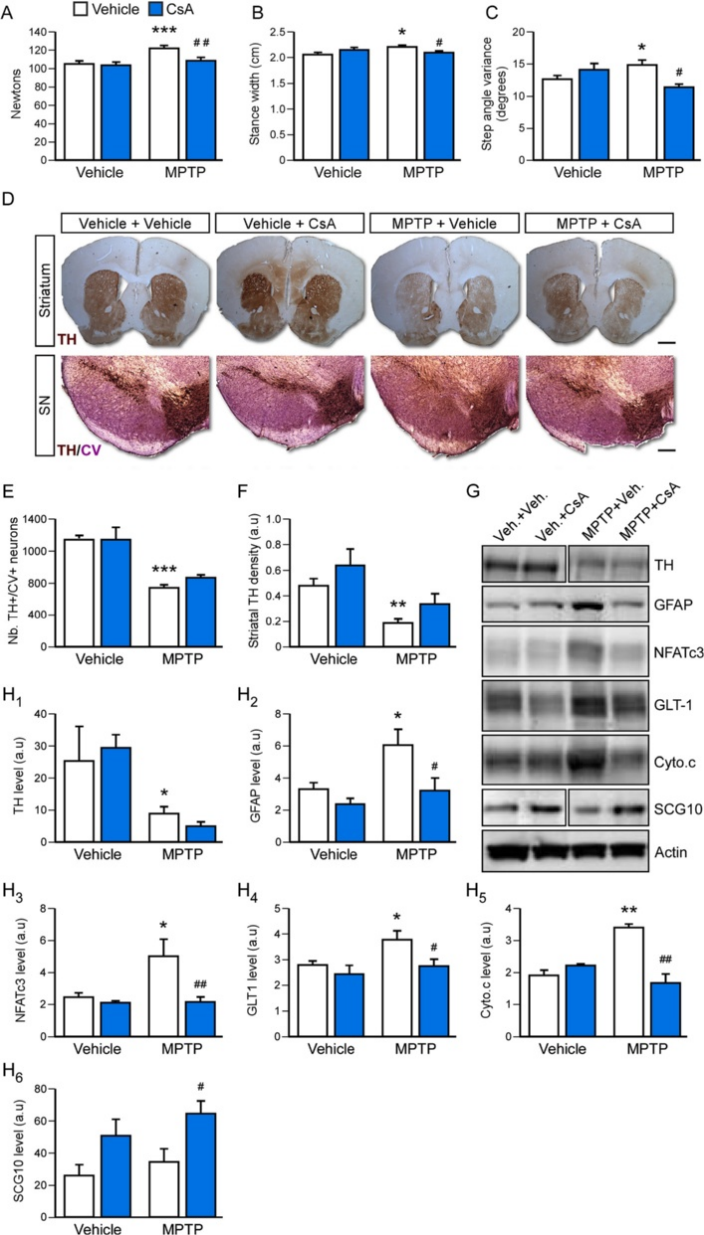
Cyclosporine alleviates motor deficits in the MPTP mouse model of PD. **a** Strength was analyzed using the grip test. Data are expressed as mean ± SEM (*n* = 5-8 per group). ***P < 0.001 compared to Veh. + Veh. group, ^##^ *P* < 0.01 compared to MPTP + Veh. group (one-way ANOVA). **b**-**c** Modifications in gait pattern were examined using the DigiGait apparatus by measuring the stance width (**b**) and the step angle variance (**c**) Data are expressed as mean ± SEM (*n* = 5-8 per group). *P < 0.05 compared to Veh. + Veh. group, ^#^ *P* < 0.05 compared to MPTP + Veh. group (one-way ANOVA). **d** Striatal and midbrain sections from all experimental groups were stained for TH and TH + Cresyl violet (CV), respectively. Scale bar: 500 μm for striatal sections and 250 μm for midbrain sections. **e** Quantification of the number of nigral TH+/CV+ dopamine neurons. Data are expressed as mean ± SEM (*n* = 5-8 per group) ***P < 0.001 compared to Veh. + Veh. group (one-way ANOVA). **f** Optical densitometry analysis of striatal TH+ fibers. Data are expressed as mean ± SEM (*n* = 5-8 per group) ** *P* < 0.01 compared to Veh. + Veh. group (one-way ANOVA). **g**-**h** Western blot analysis of TH (striatum), GFAP (striatum), NFATc3 (midbrain), GLT-1 (striatum), cytochrome c (midbrain), SGC10 (striatum) expression levels. Quantification for each marker is presented as mean ± SEM (*n* = 5-8 per group) (H_1_-H_5_). *P < 0.05 and **P < 0.01 compared to Veh. + Veh. group, ^#^ *P* < 0.05 and ^##^ *P* < 0.01 compared to MPTP + Veh. group (one-way ANOVA) (a.u: arbitrary units)

Previous studies have shown that CsA can prevent the neurodegeneration induced by MPP+ in vitro and by 6-OHDA in rodents [38, 40, 49]. In an attempt to extend our observations made in the α-synuclein-based models, we investigated whether CsA could also afford a restorative effect in this model as well. Mice received increasing doses of MPTP over 4 weeks and were treated with CsA for 4 weeks starting 3 days after the last MPTP injection. Behavioral analysis showed that CsA treatment alleviated the gripping phenotype (Fig. 6a, *P* < 0.01) and corrected the gait deficits (Fig. 6b, Ps < 0.05 compared to MPTP + vehicle group). However, examination of the integrity of the nigrostriatal pathway by counting of nigral dopamine neurons (Fig. 6d-e), densitometric measurement of striatal TH+ fibers (Fig. 6d, f) and analysis of the striatal level of TH protein (Fig. 6h) (Ps > 0.05) did not support a direct involvement of the dopaminergic system in this therapeutic effect. However, the increased expression of the axonal sprouting marker SCG-10[47] (Superior Cervical Ganglion 10) suggested that the drug may have had an effect on axonal regeneration (Fig. 6h; *P* < 0.05). Since SCG-10 is a ubiquitous marker of axonal sprouting, it cannot be determined which types of striatal projections underwent CsA-induced plasticity.

Western blot analysis confirmed the anti-inflammatory action of CsA as increased levels of NFATc3, a direct target of calcineurin regulating the expression of inflammatory factors, in the midbrain and GFAP, a marker of astrogliosis, in the striatum were observed in the MPTP + vehicle group (Ps < 0.05 compared to vehicle + vehicle group) and normalized in the MPTP + CsA group (Fig. 6h; *P* < 0.01 for NFATc3 and *P* < 0.05 for GFAP compared to the MPTP + vehicle group). MPTP-induced mitochondrial stress in the midbrain, was also assessed using the level of cytochrome c, a marker of reactive oxygen species production, and was also alleviated by CsA treatment (Fig. 6h; *P* < 0.01). MPTP lesions can also provoke an elevation of striatal GLT-1 (Glutamate Transporter1, expressed by astrocytes) suggesting increased levels of glutamate, which can modulate the activity of the striatal medium spiny neurons and possibly also induce excitotoxicity. Again we found that CsA treatment was able to dampen this deleterious event (Fig. 6h; *P* < 0.05).

### Mechanisms involved in the restorative effect of cyclosporin

Finally, we explored the possible molecular mechanisms underlying these restorative effects of CsA. Since the anti-inflammatory action of CsA could not fully account for the intra-neuronal reduction in α-synuclein levels observed in the different models, we investigated cell-autonomous mechanisms underlying the clearance of this toxic protein. In vitro experiments showed that neuronal autophagy was activated following CsA treatment as shown by the increased levels and conversion of the marker LC3II, and dephosphorylation of the autophagy-regulating protein ULK1 at residue Ser757 (Additional file 3: Supplementary Figure 2A). Activation of this degradation pathway resulted in the clearance of endogenous α-synuclein (Additional file 3: Supplementary Figure 2B).

Our observation that CsA afforded a functional recovery in the MPTP mouse model of PD without affecting the dopaminergic system while abrogating the excessive glutamatergic tone led us to hypothesize that these effects could be due to a post-synaptic mechanism. To address this question we examined the phosphorylation status of DARPP-32, a striatal-enriched phosphoprotein acting as a key hub by integrating the glutamatergic and dopaminergic signals in this tri-partite synapse. Although CsA only triggered a trend towards an increased level of phospho-Thr34-DARPP-32 in the striatum of intact and MPTP mice (*P* = 0.15 for vehicle group and *P* = 0.14 for MPTP group), a 2-way ANOVA revealed a main effect of CsA treatment on the activation of DARPP-32 [F_1,18_ = 4.68; *P* = 0.044] (Additional file 3: Supplementary Figure 2C).

## Discussion

Preclinical studies looking at therapeutic strategies in novel disease models may not only better inform their translation to the clinic, but also opens the possibility of unraveling novel pathological mechanisms which may have clinical implications in their own right. In the present study, we examined the behaviour of VM cell transplants in the recently developed AAV-α-synuclein based model of PD in comparison with the standard 6-OHDA-induced model. Although functional recovery was observed in both models, this set of experiments revealed that factors specific to the AAV-based models negatively impacted on the survival of the transplanted dopaminergic cells. Notably, we focused our attention on the immune and inflammatory reaction as (*i*) this deleterious process occurs in the human PD brain and is likely to contribute to the progression of the disease [14, 37]; (*ii*) as seen in rodent models [24, 50], a sustained immune response was reported in PD patients who underwent cell transplantation of fetal mesencephalic cells [28] and (iii) the use of immunosuppressive drugs may have influenced graft survival and efficacy in clinical trials in PD [19, 32, 42, 58]. Despite this knowledge and the well-documented pro-survival effect of CsA on dopamine progenitors grafted in the 6-OHDA model of PD [7, 11], the use of immunosuppression in the context of cell therapy in for PD patients has not been studied. In the open-label study, which demonstrated clinical benefit, patients were given immunosuppressive treatment including CsA for at least 12 months [25, 32, 58]. In contrast, in the two double-blind controlled trials that failed to show efficacy, patients were either not immunosuppressed [19] or CsA was withdrawn 6 months post-transplantation [42]. In the latter case, the slope of the grafts effects was reported to have regressed after discontinuation of the immunosuppressive regimen, supporting the idea that a rejection response developed and compromised the long-term benefit of the graft [42]. In the present study, we found that the striatum of rats overexpressing α-synuclein developed a marked chronic inflammatory process and that this likely constituted a hostile environment for cell engraftment and that its repression using CsA administration promoted the long-term survival VM grafts and especially the dopamine neurons. These data obtained in a novel rat model recapitulating several relevant features of the human condition speak in favor for the use of immunosuppression in clinical trials using allogeneic tissue.

Additional experiments in other α-synuclein models of PD, including a novel mouse model with temporal control of α-synuclein expression, confirmed the unexpected restorative effect of motor and cognitive function induced by CsA.

The therapeutic effect of CsA was first documented 20 years ago in the 6-OHDA- and MPTP-induced models of PD using a neuroprotective design [38, 40, 46, 49]. More recently, genetic manipulation and pharmacological inhibition of calcineurin using the immunosuppressant FK506 was shown to modulate α-synuclein toxicity and to afford neuroprotection in in vitro and in vivo models of PD [9, 20, 36, 56]. However, the cell-autonomous mechanisms mediating neuronal survival have not been described and we now show that CsA triggers autophagy and by so doing promotes the clearance of α-synuclein both in cultured neurons and in vivo.

Our experiments in the MPTP model reveal that CsA affects other relevant mechanisms such as the activation of DARPP-32 in striatal medium-size spiny neurons. DARPP-32 integrates signals from the nigral dopaminergic neurons and from the cortical glutamatergic projections [54]. While no restoration of the dopaminergic pathway was observed in the MPTP model, we reported a decrease in the striatal expression of GLT-1 back to the level of the control group following CsA treatment, reflecting a reduction in the glutamatergic tone, which could have resulted in increased activation of DARPP-32. In addition, DARRP-32 is a known target of calcineurin and CsA treatment stimulates the function of DARPP-32 by modulating its phosphorylation status [26, 41]. Notably in this experiment, mice were euthanized 48 h after the last injection of CsA to allow for washout of the drug which may explain why only the chronic effect of CsA was found in this experiments while no significant acute effect of CsA treatment within groups was observed. These mechanisms may, at least partly, contribute to the functional recovery seen in this model and possibly also in the α-synuclein-based models. CsA induced additional mechanisms such as axonal sprouting as shown by the increased striatal expression of SCG-10. Steiner and colleagues have previously reported the neurotrophic action of immunosuppressive drugs [52]; however, the absence of TH recovery in the MPTP model suggests that alternate neuronal sub-types may undergo axonal re-modeling/sprouting.

Although both CsA and FK506 repress calcineurin phosphatase activity, this indirect inhibition is mediated via their interaction with cyclophilin and FKBP12, respectively. Thus, it cannot be inferred that FK506 would exert a therapeutic effect via the same pathways as described here for CsA.

No PD patients, including subjects who received a sham or imitation surgery in the cell therapy trials, have been maintained under CsA treatment or other immunosuppressive treatment long enough to determine its impact on the course of the disease. Nevertheless, in a report from the open-label Swedish transplantation trial, Hagell and colleagues (1999) described the clinical progression before and after sequential bilateral grafting in PD patients who were under immunosuppression. Interestingly, patients with unilateral transplants showed a bilateral improvement in motor function even before the second transplantation was performed, while in receipt of immunosuppressive therapy including CsA [22]. Considering our novel findings, it cannot be ruled out that the immunosuppressive treatment received by these patients, which included CsA, contributed to the clinical amelioration by triggering mechanisms similar to those observed in our pre-clinical models. This hypothesis can only be verified by a proper assessment of the therapeutic effect of CsA in PD patients.

## Conclusions

In summary, as we previously highlighted when testing the effect of the glial cell line-derived neurotrophic factor (GDNF) in the AAV-α-synuclein rat model [15, 17], Our results support the view that the preclinical α-synuclein-based models of PD may be more informative than neurotoxin-induced models for the assessment of novel experimental therapies and the re-evaluation of strategies already trialed in clinic. In the context of cell replacement therapy for PD, our study provides compelling evidence for the use of immunosuppression to achieve optimal graft survival and clinical success. Together with previous neuroprotective studies [39, 40, 49], the restorative effect of CsA documented here suggests that this agent may have a place by itself in the treatment of PD.

## Additional files

Additional file 1:
**Supplementary Figure 1.** Validation of the AAV-FlexOFF-α-synuclein vector (JPG 2639 kb) Additional file 2:
**Supplementary online material.** (DOCX 119 kb) Additional file 3:
**Supplementary Figure 2. **CsA stimulates neuronal autophagy and α-synuclein clearance (JPG 712 kb)
