# Supplementary figures and images for: Cyclosporin promotes neurorestoration and cell replacement therapy in pre-clinical models of Parkinson’s disease
Source: Acta Neuropathol Commun. 2015 Dec 14;3:84. doi: 10.1186/s40478-015-0263-6 (PMC4678733; doi:10.1186/s40478-015-0263-6)

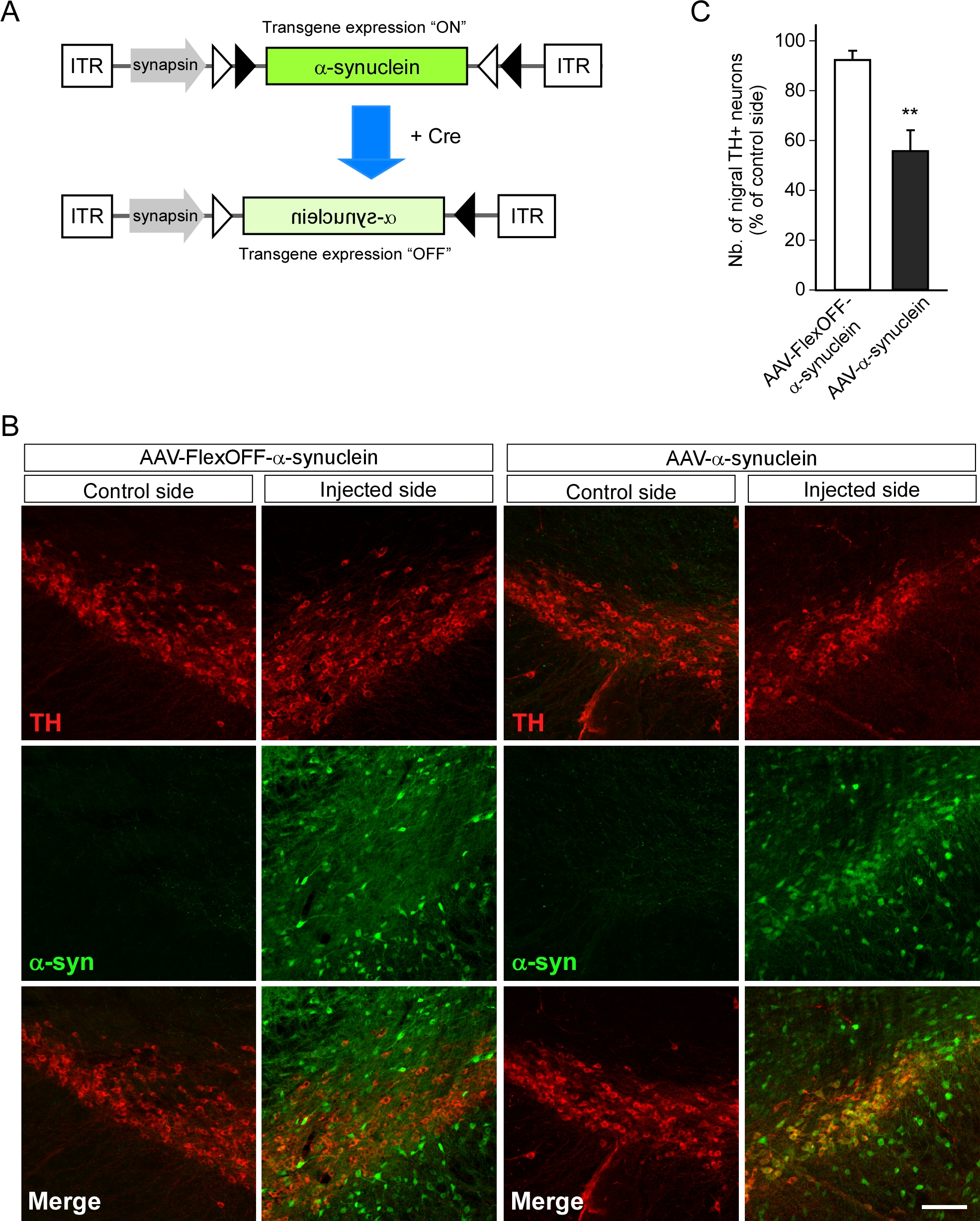

Supplement: Additional file 1: — Supplementary Figure 1. Validation of the AAV-FlexOFF-α-synuclein vector (JPG 2639 kb) [file 40478_2015_263_MOESM1_ESM.jpg]

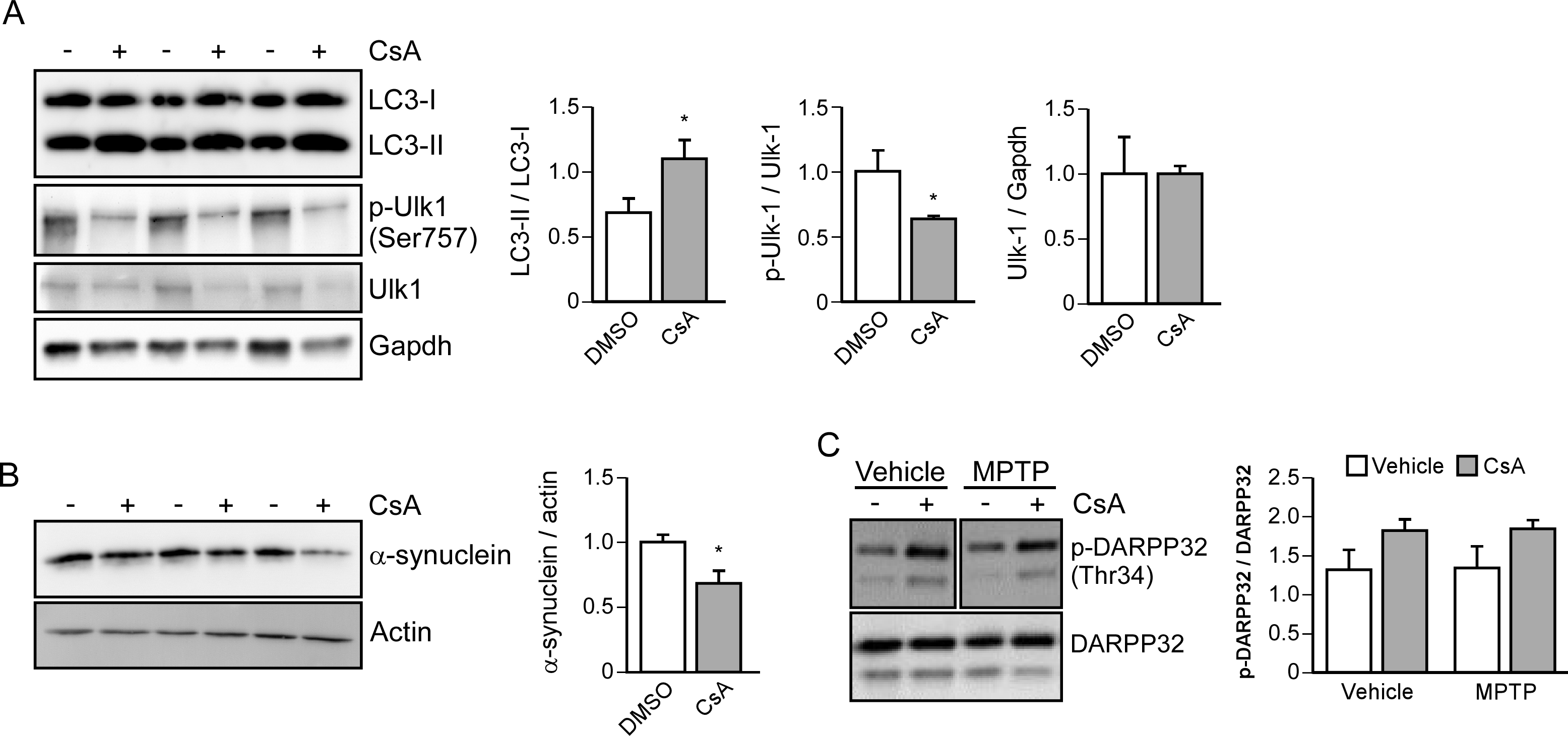

Supplement: Additional file 3: — Supplementary Figure 2. CsA stimulates neuronal autophagy and α-synuclein clearance (JPG 712 kb) [file 40478_2015_263_MOESM3_ESM.jpg]
