# Supplementary material for: Cyclosporin promotes neurorestoration and cell replacement therapy in pre-clinical models of Parkinson’s disease
Source: Acta Neuropathol Commun. 2015 Dec 14;3:84. doi: 10.1186/s40478-015-0263-6 (PMC4678733; doi:10.1186/s40478-015-0263-6)
Supplement: Additional file 2: — Supplementary online material. (DOCX 119 kb) [file 40478_2015_263_MOESM2_ESM.docx]

**Tamburrino et al. – Supplementary Material**

**SUPPLEMENTARY METHODS**

*Behavioural testing*

For the transplantation experiments, assessment of motor function was performed 1 week before the lesion, 8 weeks after the lesion (*i.e.* 1 week before the transplantation), and 6 and 12 weeks after the transplantation or sham surgery. Rats were tested for forelimb use by looking at asymmetry with the cylinder test and for amphetamine-induced rotational behaviour, as previously described (*28*).

*Cylinder test*: rats were placed in a glass cylinder (diameter 20cm), a total of 20 weight-bearing forepaw touches with the glass wall were counted, and the percentage of left (lesion-impaired) forepaw touches over total paw touches was determined.

*Rotation test*: analysis of rotations was performed in automated rotational bowls (AccuScan Instruments) coupled to the Rotameter software as described previously (*29*). Rats received an i.p injection of d-Amphetamine sulphate (2.5 mg/kg) (Apoteksbolaget, Sweden). Right and left full body turns were recorded over a period of 90 min. Data are expressed as net full turns per minute, with turns ipsilateral to the injection side given a positive value.

Eight weeks after the lesion, enrolment of animals for the transplantation procedure was based on the following threshold of motor performance in the rotation and cylinder test (*28*): >6 rotations/min and <25% of left paw use in the 6-OHDA MFB model, and >3 rotations/min and <40% of left paw use in the AAV-α-synuclein model. Rats were then randomly assigned to experimental groups (n=8-12 per group).

Thy1-α-synuclein transgenic mice and wild-type littermates were tested for motor performance using the pole test (*30*) and cognitive function was evaluated using the novel object recognition test (*31*). Tests were performed at the end of the CsA treatment.

*Pole test*: mice were placed head-up on top of a vertical wooden pole (50 cm long, 8 mm in diameter) with the base placed in the home cage. When placed at the top of the pole, mice orient themselves downward and descend into their home cage. Mice (n=10 per group) received two days of training that consisted of five trials for each session. On the test day, mice performed five trials and the total time (time to orient downward and the time to descend) was recorded. The best performance over the five trials was used for all animals (*30*)(Fleming, Salcedo et al., 2004).

*Novel object recognition*: mice were habituated to the room for one hour prior to testing. The apparatus consisted of a squared, white and opaque tank. On the first two days, mice were habituated to the arena for 5 minutes. On the third day, two identical objects were placed in the central part of the tank. Each mouse was placed in the tank and its behavior was recorded over 5 minutes. One hour later, one of the objects was replaced at random with a novel object, and the mouse´s behavior was again recorded over 5 minutes. The apparatus and objects were cleaned between subjects. The discrimination index was calculated using the following formula: *(N-F)/(N+F)*, where *F* and *N* represent exploration times (touching or sniffing) of familiar and novel objects, respectively (*31*).

Motor function was assessed in Dat-Cre^ERT2^ mice by the hanging wire test 2 days before AAV-FlexOFF-α-synuclein injection, 8 weeks after AAV vector injection and 12 weeks after CsA treatment.

*Hanging wire test*: the mice were placed on the top of a standard wire cage lid. The lid was lightly shaken to cause the animals to grip the wires then turned upside down and suspended at a height of 40 cm over an open cage filled with bedding and excess nesting materials to prevent injury from falling. The latency of mice to fall off the wire grid was measured and average values were computed from two trials (15 min apart). Trials were stopped if the mouse remained on the lid after 5 min (*19*).

*For the MPTP experiment,* Mice were tested for grip strength and gait pattern, 3 days after the last cyclosporine/vehicle injection as previously described (*32*). Using the Mesh Pull Bar attachment, mice were handled by the tail so that only the forepaws could touch the apparatus. When the forepaws were clearly latched to the Mesh Pull Bar, the mouse was slowly and steadily pulled away from the apparatus exactly parallel from the counter top until the mouse released its forepaws from the Mesh Pull Bar. Each animal’s grip strength was an average of five grip strength tests and animals were given a 5 minutes rest in between each trial. The protocol was repeated additionally, as explained above, for all the paws latching on the Mesh Pull Bar together. Grip strength was measured in Newtons.

*Gait analysis*: the running pattern was analyzed using the DigiGait apparatus (Mouse Specifics, Quincy, MA, USA) as previously described (*32*). Ventral plane videography captured the gait of each mouse through a transparent, motor-driven treadmill belt. Digital images of the paws of each mouse were taken at 150 frames/sec as mice ran at a speed of 24 cm/sec. The area of each paw relative to the treadmill belt at each frame was used for spatial and temporal measurement.

*Tissue processing and immunohistochemistry*

Animals were deeply anesthetized with i.p. injection of sodium pentobarbital (Apoteksbolaget, Sweden) and were then perfused through the ascending aorta with saline (0.9% w/v) at room temperature, followed by ice-cold paraformaldehyde (4% w/v in 0.1M phosphate buffered saline). The brains were removed, post-fixed for 2 hours in 4% paraformaldehyde and cryoprotected overnight in sucrose (25% w/v in 0.1 M phosphate buffered saline) before being sectioned on a freezing microtome or cryostat (Leica). Coronal sections were collected at a thickness of 35 µm.

Immunohistochemical stainings were performed using antibodies raised against tyrosine hydroxylase (TH) (rabbit, 1:1500; Chemicon), Iba-1 (rabbit, 1:2000; Wako), human α-synuclein (syn211, mouse, 1:2000; Santa-Cruz), synaptophysin (rabbit, 1:1000; Abcam), microtubule-associated protein 2 (MAP2) (chicken, 1:1000; Abcam) or Glial Fibrillary Acidic protein (GFAP) (rabbit, 1:2000; Abcam). Sections were rinsed three times in potassium-phosphate buffer (KPBS) between each incubation period. All incubation solutions contained 0.25% Triton X-100 in KPBS. The sections were quenched for 10 minutes in 3% H_2_O_2_/10% methanol. One hour of pre-incubation with 5% normal goat serum was followed by incubation overnight with the primary antibody in 2% serum at room temperature and incubation with the appropriate biotinylated secondary antibody (1:200; Vector Laboratories, Burlingame, CA), followed with avidin-biotin-peroxidase complex (ABC Elite; Vector Laboratories, Burlingame, CA), and visualized using 3,3-diaminobenzidine (DAB) as a chromogen, mounted and coverslipped using the DPX mounting medium.

For immunofluorescent staining, secondary antibodies coupled to Alexa 488 or Alexa 568 (1:300; Molecular Probes, Invitrogen) were used and sections were coverslipped using the FluorSave (Millipore) or the Vectashield hard set (Vectorlabs) mounting media. Images were captured using a Zeiss LSM700 confocal microscope.

For the MPTP experiments, animals were euthanized by transcardiac perfusion with 2.5% glutaraldehyde, 0.5% paraformaldehyde and 0.1% picric acid in 0.1M phosphate buffer and then post fixed in same fixative for one hour using a PELCO Biowave^R^ (Ted Pella, Redding, CA, USA), as previously described (*32*). Immunohistochemistry was performed as previously described (*32*). Briefly, coronal sections were cut through the striatal and midbrain regions at 60 μm and 40 μm respectively. The following incubations were carried out in PELCO Biowave^R^ (Ted Pella, Redding, CA, USA). Sections were subjected to an antigen retrieval step (sodium citrate, pH 6.0) for 5 minutes under vacuum and then quenched in 0.3% H_2_O_2_ in PBS for 1 minute. Sections were rinsed with PBS after each incubation. Antibodies against TH (1:400 for SN, 1:250 for striatum; Immunostar) were used. Before the primary incubation, sections were incubated in 2.5% Triton-X for 5 minutes, then placed into primary antibody for 36 minutes. A biotinylated secondary antibody, goat anti-mouse (1:100, Jackson ImmunoResearch Laboratories, West Grove, PA) was incubated for 16 minutes, followed by a 16 minute incubation with avidin-biotin-perioxidase complex (Vector, Burlingame, CA) and visualized using DAB.

*Cell counting and optical densitometry analysis*

Assessment of the total number of TH+ neurons in the SN was made according to the optical fractionator principle, using the Olympus Denmark A/S (Albertslund, Denmark) CAST-Grid system, as described previously (*33*). Every 6th section covering the entire extent of the SN was included in the counting procedure. A coefficient of error of < 0.10 was accepted. For the MPTP experiment, counting of nigral dopamine neurons was performed as previously described (*32*). A similar method was used to estimate the number of TH+ cells in the transplant and the total number of surviving dopamine cells per transplant was estimated using the Abercrombie formula (*27*).

Striatal density of dopaminergic fibers was analyzed by optical densitometry measurement using the ImagePro software.

*Western blot*

Brains were rapidly removed, and the ipsi- and contra-lateral striata were dissected and snap-frozen on dry ice. Striatal tissue and primary cortical neurons were homogenised in RIPA buffer (Sigma) with phosphatase inhibitor and protease inhibitor cocktail 2 (1:100; Sigma). Protein concentration was determined using the DC protein assay kit (Bradford method). 20 to 30 µg of protein were boiled at 90°C for 5 min in Laemmli buffer (Biorad), separated on a SDS-PAGE gel and then electrotransferred (90V, 1 hour) on a PVDF membrane (Bio-Rad). After blocking for 1 h in Tris-buffered saline with 0.1% Tween-20 (TBST) and 3% non-fat dry milk, membranes were incubated overnight at 4ºC with one of the following primary antibodies: TH (mouse, 1:2000; Immunostar), human α-synuclein (rabbit, 1:1000; Santa-Cruz), α-synuclein (rabbit, 1:1000; BD Bioscience), GAPDH (rabbit, 1:3000; Cell Signaling), actin (mouse, 1:2000; Sigma), microtubule-associated protein 1A/1B-light chain 3 (LC3) (rabbit, 1:1000; Novus Biologicals), unc-51-like kinase 1 (ULK-1) (rabbit, 1:1000; Cell Signaling), phospho-Ser757-ULK-1 (rabbit, 1:1000; Cell Signaling). After washing for 30 min in TBST with gentle agitation, membranes were incubated for 1 hour at room temperature with an HRP-conjugated secondary antibody (1:1000; Invitrogen). Protein expression was revealed using the Clarity kit (Biorad). Signal was detected using the ChemiDoc-it**^®^** Imager (UVP) and band intensities were quantified by densitometry using ImageJ v1.48 software (National Institute of Health, Bethesda, MD, USA).

For the MPTP experiments, animals were euthanized by cervical dislocation and the midbrain and dorsolateral striatum were dissected from fresh tissue. Tissue was then processed for western blots as previously described (*30*). Briefly, tissue was homogenized in lysis buffer, consisting of 5% 1.0M Tris, 2% 0.5M EDTA, 1% Triton-X 100 and 0.5% Protease inhibitor cocktail (Calbiochem, USA). 10ug/ul of protein was boiled at 95° for 5 minutes in XT Reducing Buffer with 0.5% XT reducing agent, (1:10; BioRad, Hercules, CA), then separated on 4-12% Bis-Tris XT precast gels (Biorad) at 200V for one hour, then transferred to PVDF membrane (Millipore) for 1 hour at 100V. After blocking for 1 hour in 5% dry milk in TBST, membranes were either incubated overnight at 4°C or for one hour at room temperature. Antibodies against TH (mouse, 1:40.000; Immunostar), Superior Cervical Ganglion 10 (SCG10) (1:1000; Abcam), Nuclear Factor of Activated T-cells 3 (NFATc3) (1:1000; Santa-Cruz), cytochrome c (1:1000; Santa-Cruz), Glial Fibrillary Acidic Protein (GFAP) (1:5000; Sigma), Glutamate Transporter 1 (GLT-1) (1:1000; Santa-Cruz), dopamine- and cAMP-regulated phosphoprotein, *M_r_ 32* (DARPP-32) (rabbit, 1:1000, Cell Signaling), phospho-Thr34-DARPP-32 (rabbit, 1:1000, Cell Signaling) and β-actin (1:6000, Sigma) were probed for. After membranes were rinsed in 3 times for 5 minutes and incubated in biotinylated secondary antibodies for 1 hour (anti-mouse, 1:6500; and anti-rabbit, 1:5000; Biorad). The signal was detected using Enhanced ChemiFluoresence substrate (GE Healthcare, Piscataway, NJ, USA). Visualization and quantification of the signal were performed using the UltraLum imaging system and Ultraquant 6.0 software, respectively.

**SUPPLEMENTARY FIGURES**

**Supplementary Figure 1: Validation of the AAV-FlexOFF-α-synuclein vector**

**A**: Schematic representation of the genetic construction and Cre-dependent inactivation of α-synuclein expression. The design results in “flipping” and inactivation of the transgene exclusively in Cre-expressing cells.

**B**: Midbrain sections stained for human α-synuclein (green) and TH (red) 3 weeks after intra-nigral injection of an AAV-FlexOFF-α-synuclein vector or an AAV-α-synuclein vector in Dat-IRES-Cre mice. The absence of co-localization between the two signals in the left panel confirms the inactivation of the transgene specifically in the dopamine neurons. Scale Bar: 150μm.

**C**: Stereological quantification of the number of TH+ nigral neurons shows that delivery of the AAV-FlexOFF-α-synuclein in Dat-IRES-Cre mice does not induce degeneration of dopamine neurons. Data are expressed as mean ± SEM (n=6 per group) ** P<0.01 (*t*-test).

**Supplementary Figure 2: CsA stimulates neuronal autophagy and DARPP32 function.**

**A**: Primary cortical neurons were treated with CsA (6μM) or DMSO for 24 hours. Expression levels of LC3, phospho-Ulk1(Ser757), and α-synuclein was examined by western blot. *P<0.05, t-test (mean ± SEM).

**B**: Western blot analysis of endogenous α-synuclein in primary of cortical neurons treated for 24 hours with DMSO or CsA (10μM).
